# Characterization of Mannitol-2-Dehydrogenase in *Saccharina japonica*: Evidence for a New Polyol-Specific Long-Chain Dehydrogenases/Reductase

**DOI:** 10.1371/journal.pone.0097935

**Published:** 2014-05-15

**Authors:** Zhanru Shao, Pengyan Zhang, Qiuying Li, Xiuliang Wang, Delin Duan

**Affiliations:** 1 Institute of Oceanology, Chinese Academy of Sciences, Qingdao, China; 2 University of the Chinese Academy of Sciences, Beijing, China; University of Alberta, Canada

## Abstract

Mannitol plays a crucial role in brown algae, acting as carbon storage, organic osmolytes and antioxidant. Transcriptomic analysis of *Saccharina japonica* revealed that the relative genes involved in the mannitol cycle are existent. Full-length sequence of mannitol-2-dehydrogenase (M2DH) gene was obtained, with one open reading frame of 2,007 bp which encodes 668 amino acids. Cis-regulatory elements for response to methyl jasmonic acid, light and drought existed in the 5′-upstream region. Phylogenetic analysis indicated that SjM2DH has an ancient prokaryotic origin, and is probably acquired by horizontal gene transfer event. Multiple alignment and spatial structure prediction displayed a series of conserved functional residues, motifs and domains, which favored that SjM2DH belongs to the polyol-specific long-chain dehydrogenases/reductase (PSLDR) family. Expressional profiles of *SjM2DH* in the juvenile sporophytes showed that it was influenced by saline, oxidative and desiccative factors. SjM2DH was over-expressed in *Escherichia coli*, and the cell-free extracts with recombinant SjM2DH displayed high activity on D-fructose reduction reaction. The analysis on SjM2DH gene structure and biochemical parameters reached a consensus that activity of SjM2DH is NADH-dependent and metal ion-independent. The characterization of SjM2DH showed that M2DH is a new member of PSLDR family and play an important role in mannitol metabolism in *S. japonica*.

## Introduction

Mannitol (C_6_H_14_O_6_) is one of the most abundant sugar alcohols in nature. It exists in a wide range of organisms: bacteria [1], fungi [2], [3], higher plants [4] and algae [5]. Mannitol acts as an antioxidant [6], source of reducing power and osmoregulation substance [7]. Similar to sucrose in higher plants, mannitol was proved to be a major primary photosynthetic product in *Laminaria* sp. [8], [9], *Fucus vesiculosus* and *Eisenia bicyclis* [10].

Mannitol metabolism is one of the main traits that distinguish brown algae from other phyla. In vesicular plants, the mannitol synthesis from fructose-6-phosphate is catalyzed by mannose-6-phosphate isomerase (M6PI, EC5.3.1.8), mannose-6-phosphate reductase (M6PR, EC1.1.1.224) and mannitol-1-phosphatase (M1Pase, EC3.1.3.22) [11]. While in algae, bacteria and fungi, mannitol cycle is proposed to be mediated by four enzymes: mannitol-1-phosphate dehydrogenase (M1PDH, EC1.1.1.17) and M1Pase for synthesis of mannitol and, mannitol-2-dehydrogenase (M2DH) and fructokinase (FK) for its degradation [1], [12], [13].

So far, the molecular knowledge on mannitol metabolism in algae is essentially uncharacterized. Based on expressed sequence tag (EST) libraries, Moulin et al. (1999) [14] proposed a schematic representation of carbon uptake and fixation in *Laminaria digitata*, in which mannitol metabolism was involved. With the deciphering of *Ectocarpus siliculosus* genome [15], mannitol metabolic pathway was illustrated from the points of evolution [16], metabolic analysis [17], and functional gene characterization [18], [19]. Nevertheless, except for *EsM1PDH* and *EsM1Pase*, no other reports on the molecular mechanism of mannitol cycle were addressed in brown algae so far.

Mannitol is widely applied in medicine, food and chemical industries [20] and its global market is more than 13.6 million kg/y [21]. Generally, it accounts for 10–20% (dry weight) in brown algae depending on different harvesting time [22]. In China, the mannitol yield is mainly from the kelp and other resources with annual output of approximately 8,000 t. In order to explore the mechanism of mannitol metabolism in the kelp, we initiate the study on the key enzyme of M2DH in the mannitol cycle. It is expected to decipher the structure-function relationship of SjM2DH and further benefit the yields and application of mannitol from *S. japonica* with biotechnical improvements in the future.

## Materials and Methods

### Ethics Statement

The algal samples were collected with permits and approvals of Shandong High Green Aquatic Products Co., Ltd. The sampled materials were cultivated *S. japonica* which was not protected species.

### Preculture and Treatment of *S. japonica*

Juvenile sporophytes (2–3 cm in width and 15–25 cm in length) were collected from cultivated rafts in Dec. 2012, Rongcheng, Shandong. The robust samples were selected and rinsed with filtered seawater for 3 times, and then precultured in sterilized seawater enriched with 11.76 µmol L^−1^ NaNO_3_ and 7.35 µmol L^−1^ KH_2_PO_4_ at 12°C under a photon flux density (PFD) of 45 µmol m^−2^ s^−1^.

To detect the influences of abiotic factors on the juvenile sporophytes, the samples were cultured under various salinity conditions (0‰, 8‰, 16‰, 24‰, 32‰), NaCl concentrations (400 mM, 600 mM, 800 mM, 1000 mM, 1200 mM), and H_2_O_2_ concentrations (0.2 mM, 0.4 mM, 0.6 mM, 0.8 mM, 1.0 mM). For desiccation treatments, the juvenile sporophytes were exposed in the air at 50 µmol m^−2^ s^−1^ irradiation for 0 to 4 h respectively. Generally, about 0.2 g (wet weight) samples were frozen in liquid nitrogen immediately for RNA extraction. Three independent biological replicates were performed.

### Preparation of cDNA and Total Protein Extracts

Total RNA extraction and synthesis of the first strand cDNA were performed as described by Shao et al. (2013) [23]. The extraction of total proteins from *S. japonica* was conducted according to the method reported by Rousvoal et al. (2011) [18]. The algal sample was ground in liquid nitrogen, and about 0.2 g powder was homogenized with 2 ml of lysis buffer (25 mM Tris-HCl at pH 8.0) containing 15 mM EGTA, 15 mM MgCl_2_, 2 mM DTT, 0.5% PVP, and protease inhibitors. The mixture was then transferred to intermittent sonication (Scieniz, Ningbo, China) for 2 min. After the centrifugation (15,000 g, 20 min), protein concentrations in the supernatant was measured according to the Bradford method [24].

### Isolation of the Full-length cDNA of *SjM2DH*

With analysis of the *S. japonica* transcriptome database registered in the National Center for Biotechnology Information (NCBI) (Accession number GSE33853), the unigenes related with mannitol cycle were re-verified with BLASTX algorithm (http://blast.ncbi.nlm.nih.gov/Blast.cgi). It revealed that Unigene21530 was highly homologous to M2DHs released at NCBI. Two specific primers SjM2DH-3 (5′-GCCATGGCGAACCCTCTCATTTCGGGTTTC-3′) and SjM2DH-5 (5′-CGGAAGTCGGCAGCCTTCTTACGGAGC-3′) were designed to clone the full-length cDNA by RACE method. The template synthesis and PCR programs were conducted according to the manual of Clontech SMARTer RACE cDNA Amplification Kit. The amplification protocol was as follows: 94°C for 5 min, followed by 30 cycles of 94°C for 30 s, 68°C for 30 s, 72°C 3 min, and a final extension at 72°C for 10 min. PCR products were visualized on 1% agarose gel, purified with the Gel Extraction Kit (Omega Bio-Tek, Norcross, USA), and cloned into pMD-19T vector (Takara, Tokyo, Japan). The recombinant clones were verified by sequencing in both directions using primers M13-47 and RV-M (Sangon, Shanghai, China).

### Sequence Analysis of *SjM2DH*

The coding sequence and 3′-PolyA tail identification were conducted with ORF Finder (http://www.ncbi.nlm.nih.gov/gorf/orfig.cgi) and PLOYAH [25]. The cis-regulatory elements in 5′-UTR were analyzed with PlantCARE [26]. The theoretical isoelectric point (pI) and protein molecular weight (MW) were calculated using ProtParam [27], [28]. Searches for signal peptides and transmembrane domains were done by SignalP 4.0 Server [29] and TMHMM version 2.0 program [30]. Hydrophobicity and hydrophilicity were analyzed by ProtScale program [27], and the secondary structure was predicted by SOPMA [31]. SWISS-MODEL [32]–[34] and Pymol Viewer programs were applied to construct and analyze the putative spatial structure of SjM2DH protein by homology modeling.

Multiple sequence alignment was performed with program ClustalX [35]. The phylogenetic analysis was conducted using the neighbor-joining algorithm with the software of MEGA 5.2 [36]. The bootstrap consensus tree inferred from 1000 replicates was adopted [37].

### Transcriptional Profiles of *SjM2DH*

Transcriptions of *SjM2DH* were detected with real-time quantitative PCR (RT-qPCR) procedures. The two designed specific primers qSjM2DH-F (5′- GCGAGGCAGGACACTGAAGACC-3′) and qSjM2DH-R (5′- GGGACCACATCCAGCACCAAC-3′) were applied for amplifying a 185 bp amplicon. β-actin primers qActin-F (5′-GACGGGTAAGGAAGAACGG-3′) and qActin-R (5′- GGGACAACCAAAACAAGGGCAGGAT-3′) were designed as an internal control.

RT-qPCR was performed with the SYBR *Premix Ex Taq* II (Takara, Tokyo Japan) on the TP800 Thermal Cycler Dice (Takara, Tokyo, Japan). Thermal cycling protocol was: 95°C for 30 s, followed by 40 cycles of 95°C for 5 s and 58°C for 30 s. Specificity of primers was detected by relevant dissociation curve. Three independent biological replicates were carried out for each sample, and relative quantitative values were calculated by the **2^−ΔΔCt^** method [38]. All data were subjected to one-way analysis of variance (one-way ANOVA) followed by a Student's test.

### Recombinant Expression and Purification of SjM2DH

pMAL Protein Fusion & Purification System (NEB #E8200S) was applied to perform the prokaryotic expression of SjM2DH in *E. coli*. The restriction sites of *SjM2DH* sequence were analyzed with the on-line tool WatCut (http://watcut.uwaterloo.ca/watcut/watcut/template.php). Specific primers with *Nde*I and *Eco*RI excise sites were designed to amplify the ORF of *SjM2DH* gene: Sj-M2DH-pM-F (5′-CATATGATGTCGGACTTTATCGATGCGCT-3′) and Sj-M2DH-pM-R (5′-GAATTCTTAATCGCTTTTCCCGGCAACTTCG-3′). The target ORF was then subcloned into TA cloning vector pMD 19-T vector and the reconstructed plasmid DNA was extracted with ZYMO Zyppy Plasmid Miniprep Kit. The product was digested by *Nde*I and *Eco*RI and cloned into the expression vector pMAL-c5X. The recombinant plasmid was transformed into NEB Express competent cells. The positive colonies were verified through the sequencing detection, and then cultured overnight at 37°C (shaking at 160 rpm) in LB medium, which contained 100 µg/mL ampicillin. The culture solution (2 mL) was diluted (1∶100) in pMAL rich medium (200 mL) with glucose (0.2%) and ampicillin (100 µg/mL), and then incubated at 37°C until OD_600_ reached 0.5–0.7. A final concentration of 0.3 mM IPTG was supplemented to induce the expression of the fusion protein. The cells were harvested after 2 h with centrifugation at 4,000 g for 20 min at 4°C.

The cultured cells were suspended in 15 mL column buffer (20 mM Tris-HCl pH 7.5, 200 mM NaCl, 1 mM EDTA, and 1 mM NaN_3_) and placed at −20°C overnight. Thereafter, the samples were thawed in an ice-water bath and sonicated in short pulses (10 s) until the solution was clear. The supernatant was obtained through the centrifugation at 11,400 g for 40 min.

The recombinant protein (MBP-M2DH) was purified through the maltose affinity chromatography system. The column was first equilibrated with 10 column volumes (CV) of column buffer at a flow rate of 5 mL/min. The crude extract containing the fusion protein was loaded at 4 mL/min. The column was then washed with 12 CV of column buffer and the proteins were eluted with maltose solution (50 mL, 10 mM) and collected every 2 mL. Aliquots of all the fractions were then loaded on the 12% SDS-PAGE gel for the detection of fusion MBP-M2DH protein. All the positive fractions were pooled and centrifuged in Amicon Ultra-15 Centrifugal Filter Units (Millipore).

### Determination of M2DH Activity

The activity of M2DH was determined spectrophotometrically by monitoring OD_340_ value upon NAD(P)H. The M2DH reaction mixture (200 µL) contained 100 mM reaction buffer, 1 mM hydrogen donor/acceptor, 100 mM fructose/mannitol, and ∼30 µg of protein. For assay at different pH values, sodium citrate (4.5 to 6.5), Tris-HCl (7.5 to 8.5) and glycine-NaOH (9.5 to 10.5) buffers were prepared. To optimize the temperature, the reactions were performed at various conditions from 20°C to 55°C. To verify the variation of OD_340_ was exclusively caused by M2DH, un-transformed vector, boiled extracts and ddH_2_O were applied as negative control. The reaction was initiated by the addition of substrates. Measurement of M2DH activity in the algal extracts was conducted as methods described above, and each test was repeated for three times.

## Results

### Retrieval of Genes in Mannitol Cycle

**Figure 1:**
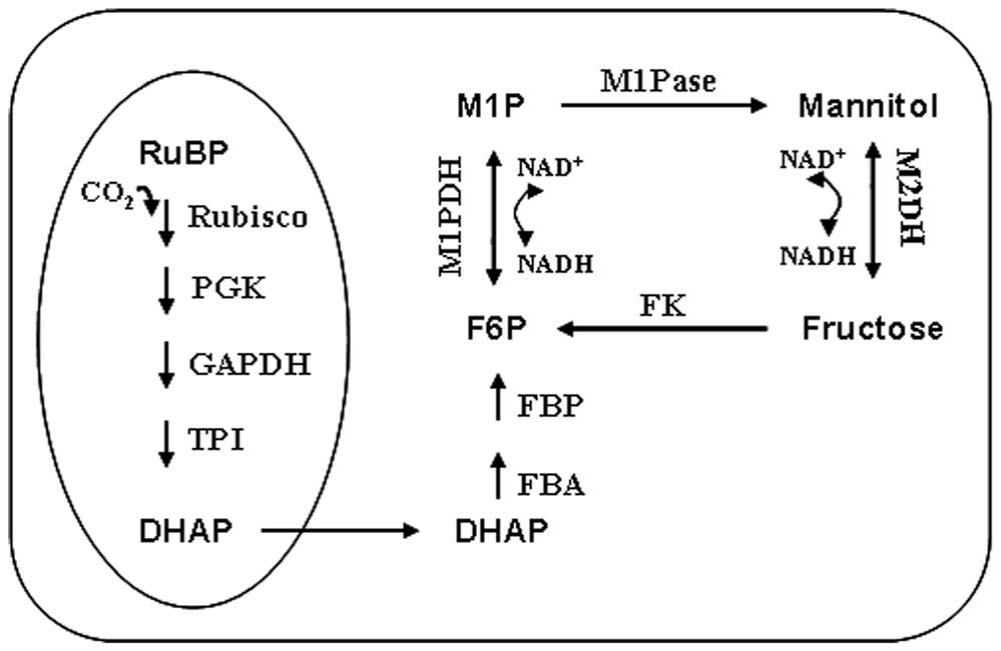
Proposed pathway for photosynthetic carbon flow to the mannitol cycle in *S. japonica*. The oval to the left represents the carbon fixation process which occurs in the chloroplast. RuBP, ribulose-1,5-bisphosphate; Rubisco, ribulose-1,5-bisphosphate carboxylase/oxygenase; PGK, phosphoglycerate kinase; GAPDH, glyceraldehyde-3-phosphate dehydrogenase; TPI, triose phosphate isomerase; DHAP, Dihydroxyacetone phosphate; FBA, fructose bisphosphate aldolase; FBP, fructose-1,6-bisphosphatase; F6P, fructose-6-phosphate; M1P, mannitol-1-phosphate; M1PDH, mannitol-1-phosphate dehydrogenase; M1Pase, mannitol-1-phosphatase; M2DH, mannitol-2-dehydrogenase; FK, fructokinase.

With KEGG enrichment analysis of *S. japonica* transcriptome, totally 8,476 unigenes were mapped to 114 pathways [39], of which, 97 unigenes (1.14%) were presumed to be involved with carbon fixation. In the annotated starch and sucrose metabolism (72 unigenes, 0.85%), mannitol cycle was retrieved. With BLASTX algorithm, 9 unigenes were verified to be related with the mannitol metabolism (Table 1), and the average length of unigenes is 1,027 bp. Based on the gene annotation, we proposed a pathway for the photosynthetic carbon flow to mannitol in *S. japonica* (Figure 1).

**Table 1:** Unigenes involved in mannitol pathway identified from transcriptome of *S. japonica* and verified with BLASTX algorithm.

Enzyme	Unigenes	Length	BlastX	Identities
Fructokinase	Unigene28398	1,167 bp	CBJ27916.1	81%
	Unigene58976	254 bp	CBJ27916.1	87%
	Unigene30536	505 bp	CBN77932.1	61%
Mannitol-1-phosphotase	Unigene5517	1,241 bp	CBN75910.1	64%
	Unigene52931	217 bp	CBJ30235.1	81%
	Unigene34062	548 bp	CBN79265.1	73%
Mannitol-2-dehydrogenase	Unigene21530	3,440 bp	CBJ29121.1	86%
Mannitol-1-phosphate	Unigene16449	1,538 bp	CBJ25895.1	87%
dehydrogenase	Unigene65528	333 bp	CBJ27644.1	88%

### Structural Characterization of *SjM2DH*

The full-length ORF of *SjM2DH* gene is 2,007 bp, encoding a protein of 668 amino acids. The length of 5′-UTR and 3′-UTR are 168 bp and 833 bp respectively. The cDNA sequence was registered in GenBank with accession numbers of KC193778.1 for mRNA sequence and AGN55416.1 for protein sequence, respectively. Cis-regulatory elements for response to MeJA (TGACG, −161 to −165), light (GGAGGG, −95 to −100) and drought (TAACTG, −78 to −83) are detected in 5′-UTR region. The predicted theoretical MW is 74.30 kDa and pI is 5.37. Neither transmembrane structures nor signal peptide were found in SjM2DH sequence, which suggested that SjM2DH are likely to be localized in the cytosol. SOPMA analysis indicated that α-helix (47.46%) and random coil (36.83%) are the major components of the secondary structures.

**Figure 2:**
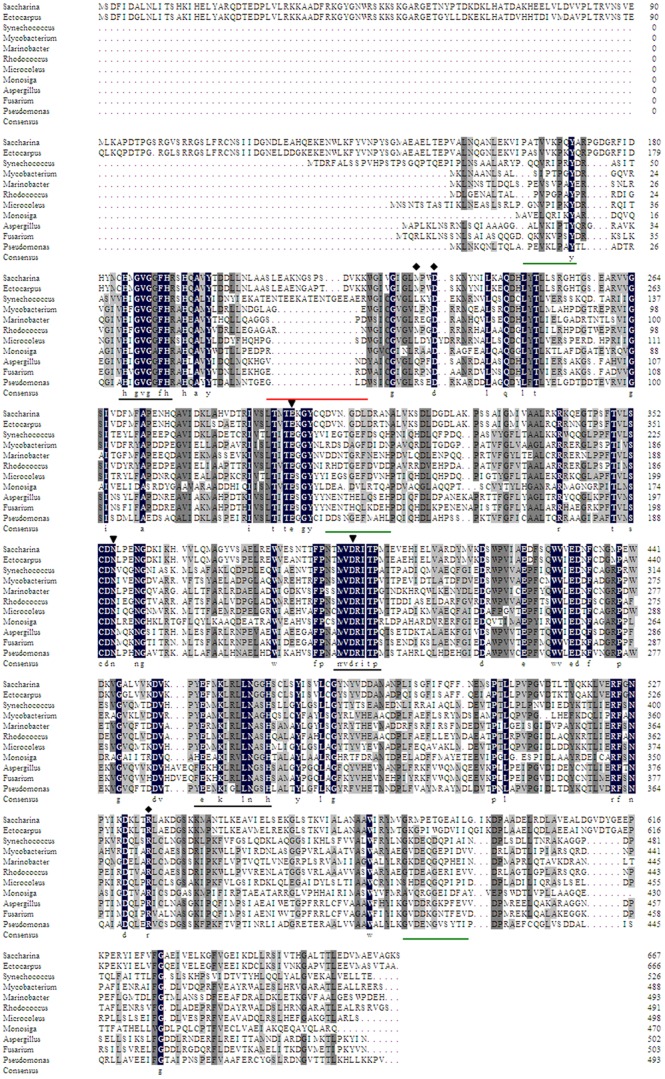
Multiple sequence alignment of MDHs from typical species of Phaeophyceae, Cyanobacteria, Actinobacteria, Proteobacteria, Ascomycota and Codonosigidae. Identical residues among all MDHs were shown in black boxes. The representative conserved regions among PSLDRs were underlined in black. The deletions of β-sheets in SjM2DH were underlined in green while the extra anti-parallel β-sheet was underlined in red. ▾, residues for substrate-binding; ⧫, residues for NADH-binding. The accession numbers were listed in File S2.

Through retrieving M2DHs protein database at NCBI, about 94.71% of M2DHs were found in bacteria and 4.92% were from fungi. BLAST alignments confirmed that SjM2DH belongs to the mannitol dehydrogenase superfamily. In brown algae, SjM2DH shared higher similarities to that of *E. siliculosus* (86% amino acid identity). Nevertheless, only ∼40% identities were found when brown algal MDHs were compared with those from bacteria, fungi and Codonosigidae (Figure S1). Multiple sequence alignment of MDHs revealed that the most conservative sequence is MVDRITP located in all the selected sequences. More than 60 conserved residues (100% identities among 14 selected species) were identified, of which one-sixth are glycines, and a conserved glycine-rich motif HxGVGxFxR (185–193 in SjM2DH) was either found (Figure 2). The adjacent conserved residues of Glu456, Lys459, Asn464 and His467 in SjM2DH sequence were identified (File S1). Accordingly, the conserved motif ExxKxxxxNxxH was examined, which was previously reported in M2DH from *Pseudomonas fluorescens* (PfM2DH) as a catalytic consensus sequence of PSLDRs [40]. Figure 2 and File S1 indicated other functional residues, most of which were identical in MDHs from Pro- and Eukaryotic species, indicating a common evolutionary origin. Except for the characteristic motifs and residues, SjM2DH contains an extension of N-terminal module (∼150 amino acids), which is unique and differs from fungi, Cyanobacteria, Proteobacteria and Actinobacteria.

### Putative Structure of SjM2DH

**Figure 3:**
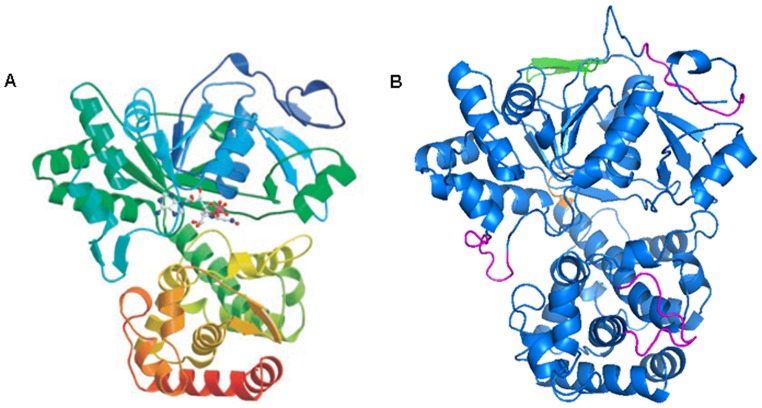
Structural comparison of the crystal structure of PfM2DH and putative SjM2DH. A, global tertiary structure of PfM2DH (Kavanagh et al., 2002). B, stereo-ribbon representation of SjM2DH in two domains. The connection of N- and C-terminal domains (VKDV) was indicated in orange; three deletions of β-sheets were shown in magenta; the insertion of an anti-parallel β-sheet was presented in green.

SjM2DH shared 39% identities of 514 amino acids to M2DH from *P. fluorescens* (AAC04472.1) (Figure S1). It is feasible to construct the tertiary structure of SjM2DH with PfM2DH as the template in SWISS-MODEL workplace. Similarly, SjM2DH folded into two domains (Figure 3), and a sequence of VKDV (Figure 3B, in orange) connects the N-terminal domain (domain 1) and C-terminal domain (domain 2). SjM2DH has a Rossmann-like fold for its catalytic activity in domain 1 with a five-stranded parallel β-sheets, flanked by six α-helices (Figure 3B). Somewhat differently, the deletion of one β-sheet (162 to 171) and two double-stranded anti-parallel β-sheets (303 to 312; 579 to 589) existed in SjM2DH structure (Figure 3B, in magenta). On the contrary, SjM2DH displayed an insertion of an anti-parallel β-sheet in the domain 1 from residue Ser209 to Val220 (Figure 3B, in green).

### Phylogenetic Analysis of M2DH Amino Acid Sequences

**Figure 4:**
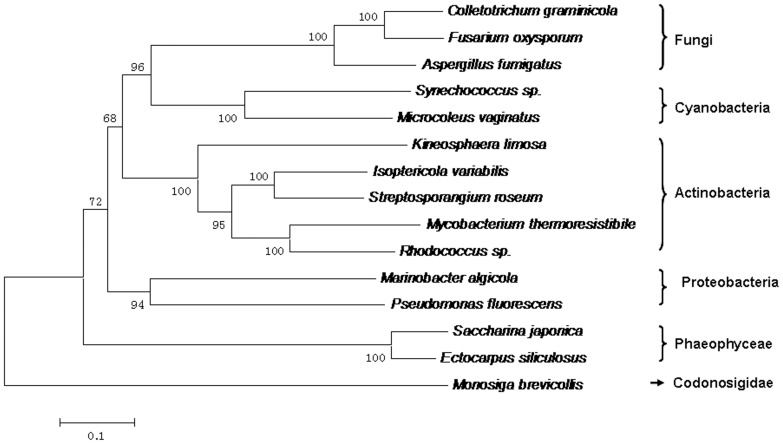
Phylogenetic tree constructed based on alignment of 15 M2DH amino acid sequences. The tree was obtained by the neighbor-joining algorithm using the MEGA 5.2 program. Bootstrap values calculated from 1,000 replicates were given. The scale bar corresponded to 0.1 estimated amino-acid substitutions per site.

For phylogenetic analysis, 9 MDHs sequences from bacteria, 3 from fungi, and 3 from Stramenopiles were aligned (File S2). Interestingly, the MDHs from fungi, brown algae and *Monosiga* were not clustered into a separate “eukaryotic” clade as expected (Figure 4). M2DHs from brown algae and fungi were nested within the bacterial clade (Cyanobacteria, Proteobacteria and Actinobacteria), and the neighbor sub-family is from Choanoflagellida. For PSLDRs proteins, M2DHs from brown algae and bacteria had a closer evolutionary history when compared to other eukaryotic species.In addition, the phylogenetic tree using the maximum likelihood (ML) method is identical with NJ tree (Figure S2).

### Transcriptional Profiles of *SjM2DH*

**Figure 5:**
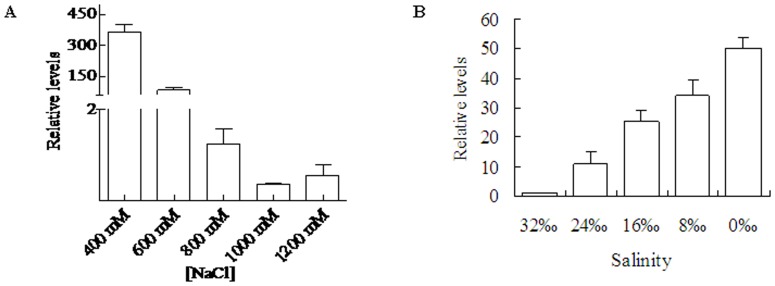
Influence of salinities and NaCl concentrations on *SjM2DH* expression levels in juvenile sporophytes. A, expression levels of *SjM2DH* under various NaCl concentrations. B, expression levels of *SjM2DH* under various salinities. All the data are the mean values of three independent experiments.

One-way ANOVA on the variation of expression of *SjM2DH* showed significant changes under NaCl treatment (F = 52.84, *P*<0.001). The detected *SjM2DH* transcriptions were relatively higher under 400 mM NaCl and decreased remarkably with increasing NaCl concentration. It displayed a 4.37-fold decrease in 600 mM and about 1000-fold decrease in 1000 mM NaCl (Figure 5A). The transcripts of *SjM2DH* increased with the salinities decreased from 32‰ to 0‰, 9.35-fold at 24‰ compared to that under 32‰ salinity. After immersing in freshwater (0‰ salinity) for 2 h, the relatively high transcriptional level appeared, and it was 43.87 times than that at 32‰ seawater (Figure 5B). Significant changes were observed during the decrease of salinity with one-way ANOVA analysis (F = 25.77, *P*<0.01).

**Figure 6:**
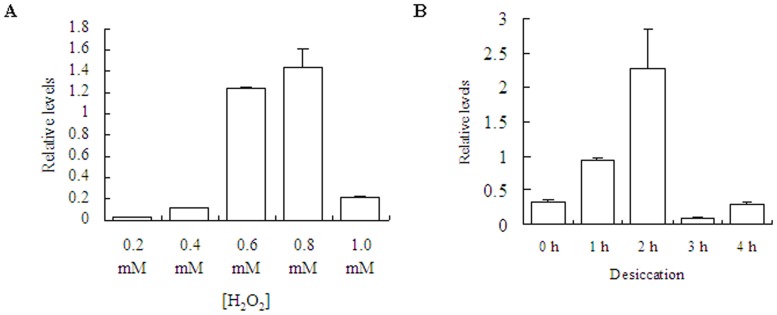
Influence of H_2_O_2_ concentrations and desiccation on *SjM2DH* expression levels in juvenile sporophytes. A, expression levels of *SjM2DH* under various H_2_O_2_ concentrations. B, expression levels of *SjM2DH* under different duration of desiccation stress. All the data are the mean values of three independent experiments.

Influence of oxidative and desiccative stress on *SjM2DH* expressions were analyzed (Figure 6). The transcriptional level was extremely low when under 0.2 mM H_2_O_2_ treatment, and gradually rose with increasing of H_2_O_2_ concentrations. It exhibited 59.51-fold increase when under 0.8 mM H_2_O_2_ compared to that of 0.2 mM (Figure 6A). With extension of drying time, *SjM2DH* relative levels reached maximum at 2 h (6.89-fold) and then decreased dramatically to 0.05 times compared with that of 2 h group (Figure 6B). After oxidative and desiccative stress, a similar trend emerged under both treatments and it was statistically significant with *P*<0.01.

### Identification of Recombinant SjM2DH

**Figure 7:**
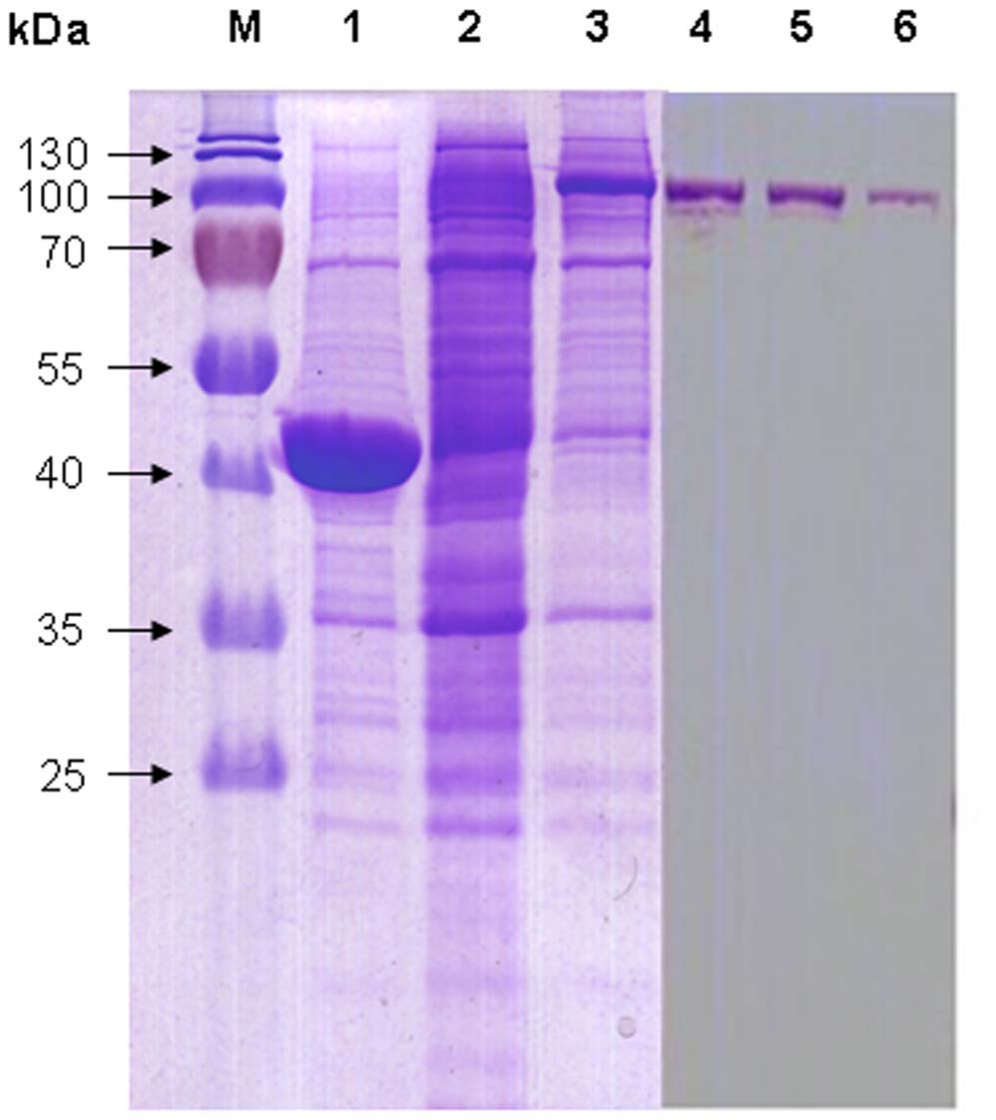
SDS-PAGE (12%) analysis of induction and purification of recombinant SjM2DH. M, protein marker; lanes 1 and 2, negative controls of untransformed vector and recombinant *E. coli* induced with 0 mM IPTG, respectively; lane 3, recombinant *E. coli* with 0.3 mM IPTG induction for 1 h; lanes 4–6, eluted fractions with presence of recombinant SjM2DH.

For the *in vitro* expression, full-length ORF of *SjM2DH* gene was cloned into pMAL-c5X vector. After the transformation to expression strain (*E. coli* NEB Express), a fusion protein MBP-M2DH was induced with 0.3 mM IPTG for 2 h and was separated on the SDS-PAGE electrophoresis (12%). pMAL-c5X vector was either transformed into expression strain with the same induction conditions as negative control. The gel displayed clear bands consistent with the predicted MW of 115 kDa for the recombinant SjM2DH (Figure 7), whereas the target bands were not observed at lanes corresponding to the control groups. The MBP-M2DH fusion protein was then eluted via maltose affinity chromatography, and the positive fractions showed a single band at MW of target protein (Figure 7).

### Enzymatic Assay of SjM2DH

**Figure 8:**
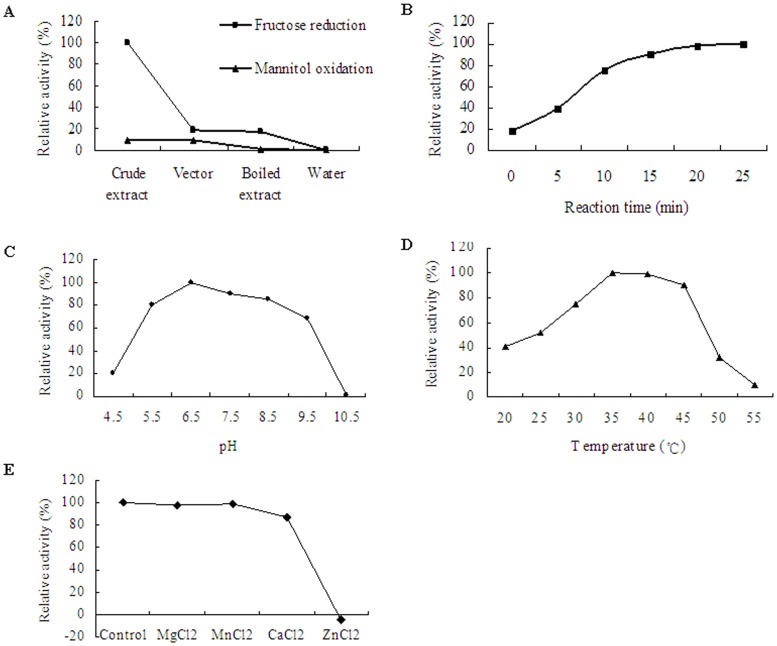
Enzymatic characteristics of recombinant SjM2DH in cell-free bacterial extracts. A, relative activity in fructose reduction and mannitol oxidation directions. B, time-course evaluation (0–25 min) of reductive activity on fructose. C, influence of different pH (4.5–10.5) on the activity of SjM2DH. D, influence of temperature (20–55°C) on SjM2DH activity. E, influence of metal ion on the activity of SjM2DH.

M2DH activity was testified at 35°C in 100 mM Tris-HCl buffer (pH 7.5). When D-fructose was applied as the substrate, a depletion of NADH was detected in crude extracts with recombinant SjM2DH (Figure 8A). However, little change of OD_340_ values were detected with NADPH as cofactor (data not shown). Compared to the control groups, no increase of OD_340_ was detected with mannitol oxidation direction, neither with NAD^+^ nor NADP^+^ as cofactor (Figure 8A). The OD_340_ values were recorded from 0 min to 25 min, and the concentration of NADH was decreased from ∼0.99 mM at the starting point to 0.17–0.19 mM after incubation for 20–25 min. The relative activity increased sharply within 10 min, and then crept up to the maximum afterwards (Figure 8B). The optimum pH for reduction by SjM2DH was 6.5, with 90.56% and 85.28% of the maximum activity at pH 7.5 and 8.5 (Figure 8C). The optimum temperature for reduction of D-fructose was between 35°C and 40°C. M2DH remained 40.92% of the maximum activity at 20°C, whereas the activity was scarcely detectable at 55°C (Figure 8D). ZnCl_2_ has been shown to inhibit M2DH activity, while the influence of MgCl_2_, CaCl_2_ and MnCl_2_ was scarcely detected (Figure 8E).

## Discussion

Mannitol metabolism in marine plants is poorly understood so far. Although carbohydrate metabolism was deduced from genomic analysis of diatoms, no molecular reports were on mannitol cycle [41], [42]. In brown algae, the limited molecular knowledge available comes from M1PDH and M1Pase enzymatic assays in *E. siliculosus* [18], [19]. With regard to M2DH, no molecular studies were conducted so far except for the release of nucleotide sequence in *E. siliculosus* (Esi0135_0025) [15], [16].

### Horizontal Gene Transfer of *M2DH* in Brown Algae

According to the biochemical characters of mannitol-producing or degrading enzymes, the mannitol pathway in algae was considered to be basically similar to that in fungi [12]. Here with the phylogenetic analysis of M2DHs, the SjM2DH was clustered into prokaryotic clade, which is closer to Proteobacteria and Actinobacteria. Although highly conservative residues were identical in Pro- and Eukaryotic species, the closer phylogenetic relationship indicated that *SjM2DH* was probably acquired from bacterial genome via horizontal gene transfer (HGT) event. This was consistent with large-scale HGT found in carbon storage and cell wall biosynthesis in *E. siliculosus* [16], [43], [44].

### SjM2DH is a New Member of PSLDR Family

Commonly, MDHs of fungi and higher plants belong to SDR [2], [3] and MDR family [45], [46], respectively. However, gene structural and phylogenetic analysis of SjM2DH favored that SjM2DH is more alike to bacterial M2DHs, which belong to PSLDR family. Unlike SDRs and MDRs needing a triad of conserved Ser-Tyr-Lys residues [47] or metal ion [48] for catalysis, a conserved Lys459 acted as the basic base for SjM2DH activity. A highly conserved KxxxxNxxH motif was verified to be a unique catalytic signature among all PSLDR members [49]. Here in this study, the presence of KLRLLNGGH segment in SjM2DH sequence is in accordance with this feature of PSLDRs.

Previously, M2DHs identified from fungi and red algae were believed to be NADP(H)-dependent [2], [50], while bacterial M2DHs (PSLDR members) exclusively use NAD(H) as cofactor [51]. Here in our study, the presence of Asp234 and absence of Arg231 contributed to the specificity for NAD(H) as cofactor over NADP(H) for SjM2DH. Accordingly, the reduction of fructose by recombinant SjM2DH exclusively uses NADH as cofactor, which favored that SjM2DH is a member of PSLDR family. However, *SjM2DH* gene encodes a protein of 668 amino acids unexpectedly, which is beyond the length of reported PSLDRs (∼360–550 amino acids) so far. After searching “mannitol dehydrogenase” in NCBI protein database, the extension of N-terminal module was exclusively found in MDHs of brown algae and did not align with the better-characterized MDHs so far. Therefore, it is believed that the specificity of N-terminal domain should have influence on SjM2DH function. The deletion and insertion of β-sheets in SjM2DH spatial structure might be another character distinguishing brown algal M2DHs. Nevertheless, it needs to verify the function of these regions in the future.

### SjM2DH Functions in Abiotic Stress Tolerance

Referred to sub-lethal stress conditions determined for *E. siliculosus* [52], we applied 400–1000 mM NaCl, 0–32‰ salinities to testify the influence of hyper- and hyposaline stress on *SjM2DH*. Short-term treatment of 2 h for each individual was adopted to avoid cell death. Unlike the up-regulation of *EsM1PDH1* and *EsM1PDH2* under hypersaline conditions [18], the transcription of *SjM2DH* decreased with increasing of NaCl concentrations. As M2DH could catalyze the mannitol oxidation, the decreasing trend implied that the kelp might resist high NaCl concentrations outside via reducing mannitol degradation. The juvenile sporophytes could maintain robust growth in the salinity as low as 0‰ for 2 h, with some “bubbles” developed owing to absorbing water from outside. Consequently, the transcription of *SjM2DH* increased significantly with salinity decreasing, which might be due to the function of fructose reduction by M2DH. It is thus presumed that the kelp might keep osmotic pressure through the regulation of the catalytic direction between mannitol and fructose.

Naturally, *S. japonica* niches in sublittoral environments, and has poor acclimation to desiccation and oxidative stress compared with intertidal algae [53], [54]. Here in this study, remarkable up-regulation of *SjM2DH* was found under desiccation for 1–2 h and 0.2 to 0.8 mM H_2_O_2_ treatment. Long duration of desiccation and higher concentration of H_2_O_2_ could not cause a significant transcription of *SjM2DH*. These results implied that mannitol metabolism might be involved in physiological process response to temperate desiccative and oxidative stress. In addition, a cis-regulatory element for drought-response exists in the upstream sequence of *SjM2DH* ORF, which could strengthen the function of M2DH under desiccation stress.

### Enzymatic Characterization of Recombinant SjM2DH

In general, mannitol dehydrogenases catalyze the reversible reaction between D-mannitol and D-fructose [40]. The catalytic characteristics of recombinant SjM2DH were determined in this study, and SjM2DH activity was only detected in the direction of fructose reduction, but not for the mannitol oxidation. Moreover, SjM2DH catalyzed fructose reduction with NADH as cofactor rather than NAPDH, which is consistent with prokaryotic MDHs [55], whereas eukaryotic fungi and yeasts are NADPH dependent [56], [57]. The preference for NADH could be explained by the presence of Met231, Asp234 and Arg536 in SjM2DH sequence.

The relative activityof SjM2DH were higher than 80% in the buffer pH from 5.5 to 8.5. In most cases, optimal pH for reduction by M2DHs is between 7.0 and 8.0 [58], [59]. Besides, 35–40°C was verified to be the optimal temperature to catalyze reduction of fructose, and this is accordance with published results from *C. leprieurii* [60]. Moreover, there is no need for Mg^2+^ and/or Ca^2+^ as activators in the catalyze site due to the existence of Lys459, since its NH_2_ group was believed to participates in a pre-catalytic equilibrium process [61]. The result that MgCl_2_ and CaCl_2_ had no activating effects on M2DH activity was in agreement with its structural characters. Unlike MDRs, M2DHs did not use Zn^2+^ in catalysis due to lack of Cys/His ligand [49], [62], which was verified by the inhibition of ZnCl_2_ on SjM2DH activity in this study.

Although pMAL expression system softened the insoluble expression problems, the detected purified recombinant M2DH exhibited no activity. It may ascribe to the long-term retention at room temperature after cell lysis. Besides, NaCl, EDTA and sodium azide may inhibit the peptide activity. In addition, endogenous M2DH activity was neither detected, which was consistent with extremely lower M2DH activity detected in *D. grisea* [58]. Considering the characterization of GDP-mannose dehydrogenase from *E. siliculosus* by constructing a His-tagged MBP recombinant plasmid with a TEV protease cleavage site [63], more explorations are needed to be further conducted.

## Supporting Information

Figure S1 **Comparison of sequence positives and identities from representative MDH amino acid sequences from each subgroup.**
(TIF)

Figure S2 **Phylogenetic tree constructed with maximum likelihood (ML) method based on alignment of 15 M2DH amino acid sequences.**
(TIF)

File S1 **Comparison among crucial amino acid residues from different M2DHs.**
(DOC)

File S2 **Accession numbers of M2DH sequences from all the selected species for phylogenetic analysis.**
(DOC)
