# Supplementary figures and images for: Characterization of Mannitol-2-Dehydrogenase in Saccharina japonica: Evidence for a New Polyol-Specific Long-Chain Dehydrogenases/Reductase
Source: PLoS One. 2014 May 15;9(5):e97935. doi: 10.1371/journal.pone.0097935 (PMC4022671; doi:10.1371/journal.pone.0097935)

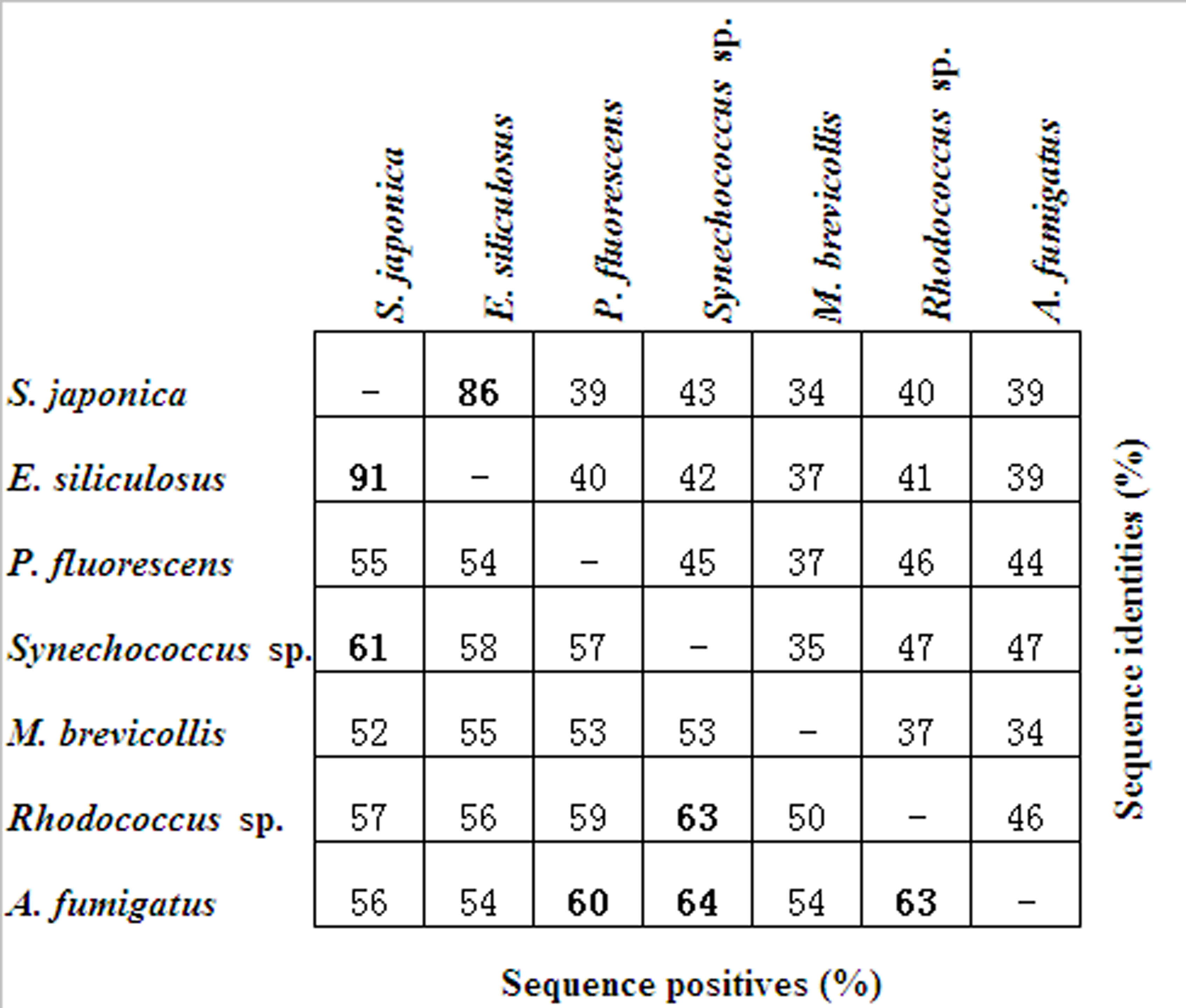

Supplement: Figure S1 — Comparison of sequence positives and identities from representative MDH amino acid sequences from each subgroup. (TIF) [file pone.0097935.s001.tif]

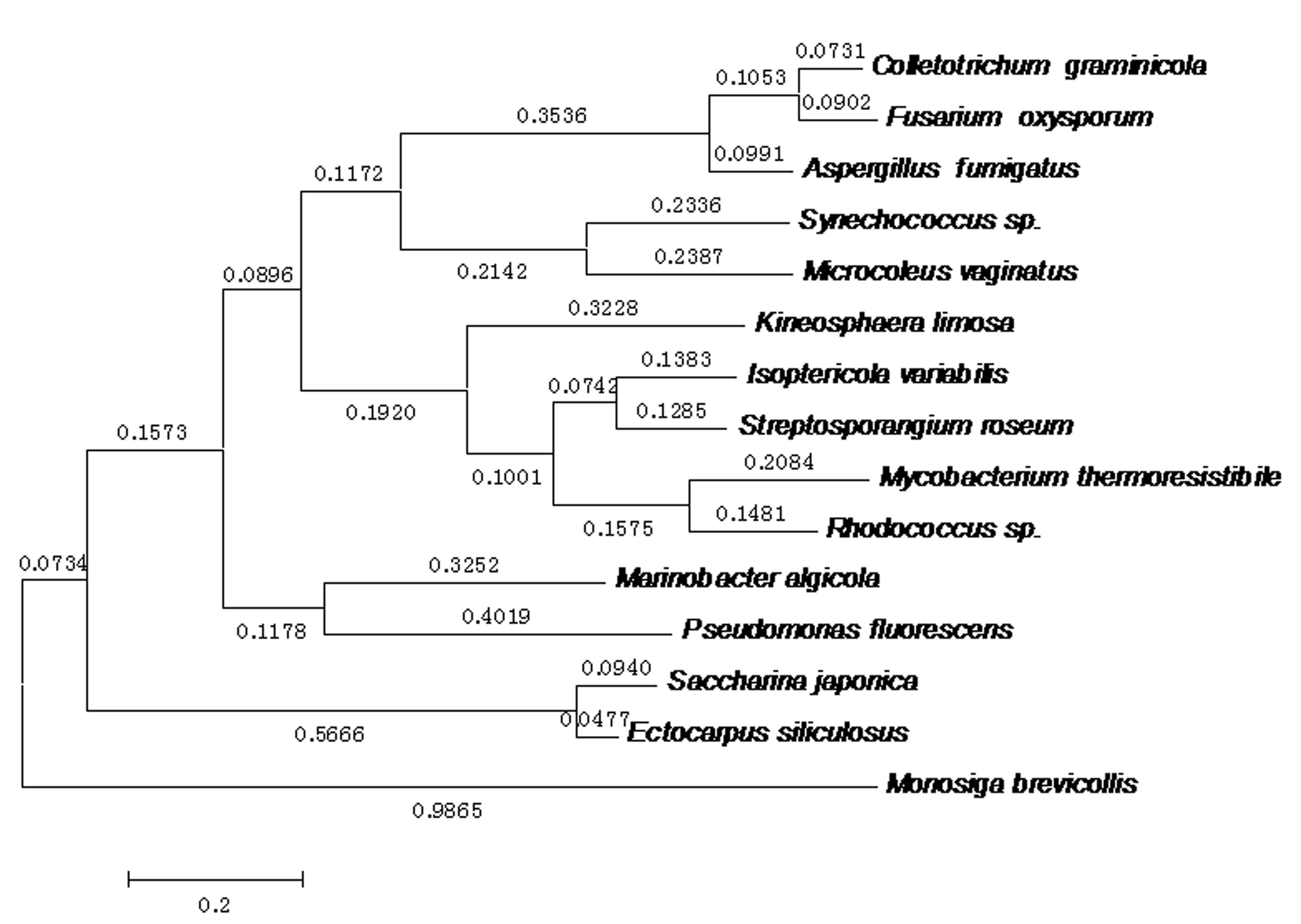

Supplement: Figure S2 — Phylogenetic tree constructed with maximum likelihood (ML) method based on alignment of 15 M2DH amino acid sequences. (TIF) [file pone.0097935.s002.tif]
