# Supplementary material for: Characterization of Mannitol-2-Dehydrogenase in Saccharina japonica: Evidence for a New Polyol-Specific Long-Chain Dehydrogenases/Reductase
Source: PLoS One. 2014 May 15;9(5):e97935. doi: 10.1371/journal.pone.0097935 (PMC4022671; doi:10.1371/journal.pone.0097935)
Supplement: File S1 — Comparison among crucial amino acid residues from different M2DHs. (DOC) [file pone.0097935.s003.doc]

File S1 Comparison among crucial amino acid residues from different M2DHs

| *S. japonica* | *E. siliculosus* | *P. fluorescens* | *Synechococcus* sp. | *Rhodococcus sp.* | *A. fumigatus* | *M. brevicollis* |
| --- | --- | --- | --- | --- | --- | --- |
| Met231 | Met230 | Arg66 | Leu104 | Met64 | Gln74 | Arg54 |
| Asp234 | Asp233 | Asp69 | Asp107 | Asp67 | Asp77 | Asp57 |
| Glu297 | Glu296 | Glu133 | Glu170 | Glu131 | Glu140 | Glu121 |
| Asn355 | Asn354 | Asn191 | Asn228 | Asn189 | Asn200 | Asn177 |
| Asp394 | Asp393 | Asp230 | Asp267 | Asp228 | Asp239 | Asp217 |
| Glu456 | Glu455 | Glu292 | Glu329 | Glu290 | Glu304 | Glu279 |
| Lys459 | Lys458 | Lys295 | Lys332 | Lys293 | Lys307 | Lys282 |
| Asn464 | Asn463 | Asn300 | Asn337 | Asn298 | Asn312 | Asn287 |
| His467 | His466 | His303 | His340 | His301 | His315 | His290 |
| Arg536 | Arg535 | Arg373 | Arg409 | Arg371 | Arg385 | Arg359 |
