# Supplementary material for: Characterization of Mannitol-2-Dehydrogenase in Saccharina japonica: Evidence for a New Polyol-Specific Long-Chain Dehydrogenases/Reductase
Source: PLoS One. 2014 May 15;9(5):e97935. doi: 10.1371/journal.pone.0097935 (PMC4022671; doi:10.1371/journal.pone.0097935)
Supplement: File S2 — Accession numbers of M2DH sequences from all the selected species for phylogenetic analysis. (DOC) [file pone.0097935.s004.doc]

File S2 Accession numbers of M2DH sequences from all the selected species for phylogenetic analysis

| **Species** | **NCBI Acc. Number** |
| --- | --- |
| *Aspergillus fumigatus* Af293 | XP_751388.1 |
| *Colletotrichum graminicola* M1.001 | EFQ34772.1 |
| *Ectocarpus siliculosus* | CBJ29121.1 |
| *Fusarium oxysporum* f. sp. vasinfectum 25433 | EXM19286.1 |
| *Isoptericola variabilis* 225 | [YP_004543375.1](http://www.ncbi.nlm.nih.gov/protein/334338223?report=genbank&log$=prottop&blast_rank=48&RID=P7C1U9CS01R) |
| *Kineosphaera limosa* NBRC 100340 | [ZP_10939830.1](http://www.ncbi.nlm.nih.gov/protein/403713746?report=genbank&log$=prottop&blast_rank=22&RID=P7C1U9CS01R) |
| *Marinobacter algicola* DG893 | [ZP_01892641.1](http://www.ncbi.nlm.nih.gov/protein/149374868?report=genbank&log$=prottop&blast_rank=81&RID=P7C1U9CS01R) |
| *Microcoleus vaginatus* FGP-2 | [ZP_08493802.1](http://www.ncbi.nlm.nih.gov/protein/334119717?report=genbank&log$=protalign&blast_rank=5&RID=P714Y912014) |
| *Monosiga brevicollis* | XP_001750640.1 |
| *Mycobacterium thermoresistibile* ATCC 19527 | [ZP_09083459.1](http://www.ncbi.nlm.nih.gov/protein/357021228?report=genbank&log$=prottop&blast_rank=40&RID=P7C1U9CS01R) |
| *Pseudomonas fluorescens* | [AAC04472.1](http://www.ncbi.nlm.nih.gov/protein/2293418?report=genbank&log$=prottop&blast_rank=1&RID=P7GZFN0T014) |
| *Rhodococcus* sp. P14 | [ZP_11103958.1](http://www.ncbi.nlm.nih.gov/protein/407275488?report=genbank&log$=prottop&blast_rank=30&RID=P7C1U9CS01R) |
| *Saccharina japonica* | KC193778.1 |
| *Streptosporangium roseum* DSM 43021 | [YP_003339175.1](http://www.ncbi.nlm.nih.gov/protein/271964979?report=genbank&log$=protalign&blast_rank=13&RID=P714Y912014) |
| *Synechococcus* sp. PCC 7335 | [ZP_05039415.1](http://www.ncbi.nlm.nih.gov/protein/254425698?report=genbank&log$=protalign&blast_rank=3&RID=P714Y912014) |
